# A framework for the evaluation of patients with congenital facial weakness

**DOI:** 10.1186/s13023-021-01736-1

**Published:** 2021-04-07

**Authors:** Bryn D. Webb, Irini Manoli, Elizabeth C. Engle, Ethylin W. Jabs

**Affiliations:** 1 Department of Genetics and Genomic Sciences, Icahn School of Medicine at Mount Sinai, New York, NY USA; 2 Department of Pediatrics, Icahn School of Medicine at Mount Sinai, New York, NY USA; 3 Medical Genomics and Metabolic Genetics Branch, National Human Genome Research Institute, National Institutes of Health, Bethesda, MD USA; 4 Department of Neurology, Boston Children’s Hospital, Harvard Medical School, Boston, MA USA; 5 Department of Ophthalmology, Boston Children’s Hospital, Harvard Medical School, Boston, MA USA; 6 Howard Hughes Medical Institute, Chevy Chase, MD USA

**Keywords:** Congenital facial weakness, Facial paralysis, Clinical genetics, Clinical characterization

## Abstract

There is a broad differential for patients presenting with congenital facial weakness, and initial misdiagnosis unfortunately is common for this phenotypic presentation. Here we present a framework to guide evaluation of patients with congenital facial weakness disorders to enable accurate diagnosis. The core categories of causes of congenital facial weakness include: neurogenic, neuromuscular junction, myopathic, and other. This diagnostic algorithm is presented, and physical exam considerations, additional follow-up studies and/or consultations, and appropriate genetic testing are discussed in detail. This framework should enable clinical geneticists, neurologists, and other rare disease specialists to feel prepared when encountering this patient population and guide diagnosis, genetic counseling, and clinical care.

**Supplementary Information:**

The online version contains supplementary material available at 10.1186/s13023-021-01736-1.

## Clinical characteristics: congenital facial weakness

**Fig. 1:**
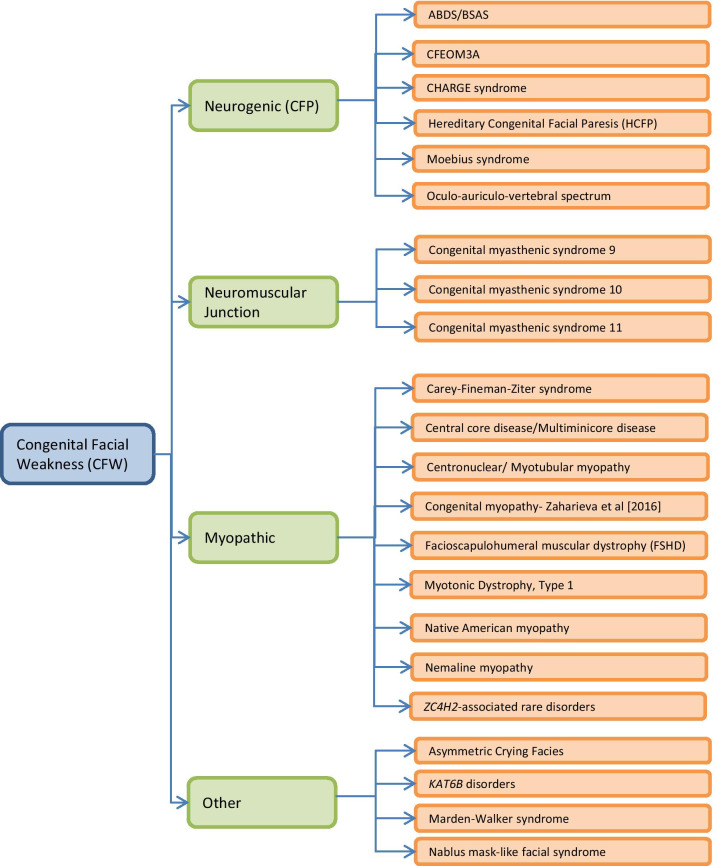
Differential Diagnosis for CFW Disorders. CFW disorders may be due to neurogenic, neuromuscular junction, myopathic, or other causes

Congenital facial weakness (CFW) refers to decreased facial movement present at birth secondary to impaired function of facial musculature. CFW may be secondary to a defect in the motor nucleus of the facial nerve or the facial nerve itself (cranial nerve 7; CN7) (neurogenic), a defect at the neuromuscular junction, an inherent muscular problem (myopathic), or other unknown or mixed causes (Fig. 1). Congenital facial paralysis (CFP) is decreased (palsy/paresis) or absent (paralysis) facial movement present at birth that results specifically from loss of facial nerve function. CFP may be caused by an abnormal developmental process or other causes, including the most common cause of trauma, as in the case of temporary or permanent CFP resulting from the use of forceps during delivery. The differential provided in this review is useful after exclusion of post-traumatic CFP. An appropriate history and temporary CFP are important considerations for diagnosis of traumatic CFP.

**Fig. 2:**
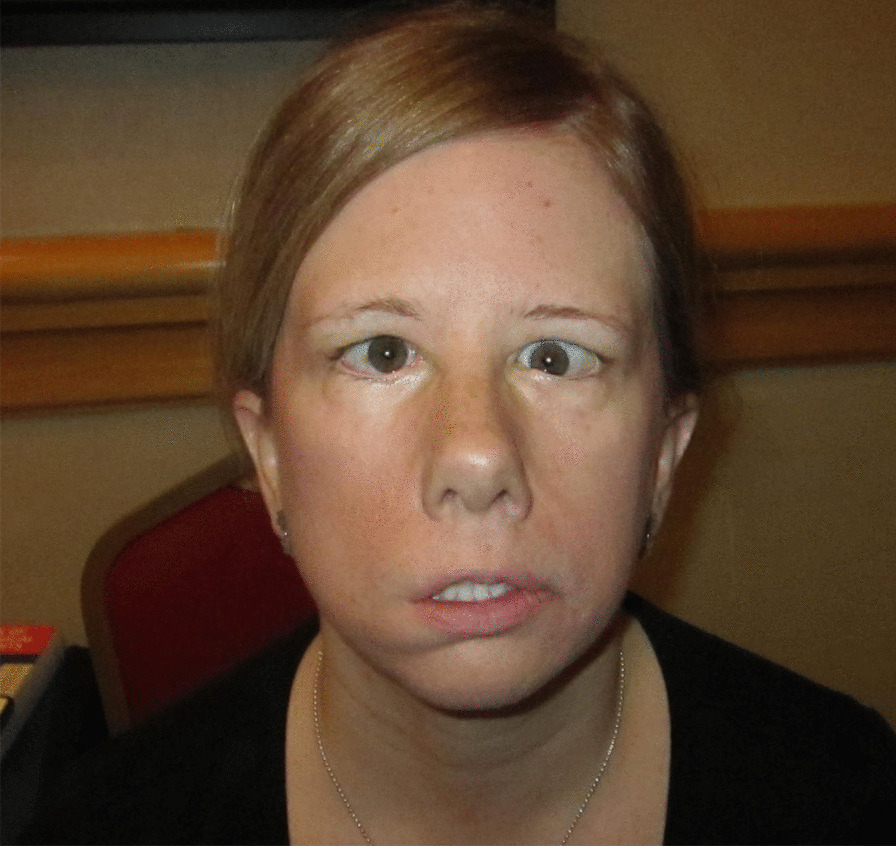
CFW seen in an adult female. This adult female with Moebius syndrome has CFW, often described as causing a “mask-like” facial appearance. She has bilateral CFP and is more affected on her right side

CFW may be unilateral or bilateral and may be partial or complete (Fig. 2). Complete CFW refers to complete absence of facial movement in all four quadrants of the face (right upper quadrant, right lower quadrant, left upper quadrant, and left lower quadrant). Patients with complete absence of facial movement on the left side of the face may be described as having unilateral (left) complete CFW. Similarly, patients with reduced facial movement on the left side of the face may be described as having unilateral (left) partial CFW. Clinical characteristics of CFW may include: facial droop; absence of forehead, nasolabial, or periorbital folds; lagophthalmos (incomplete eyelid closure); open mouth posture or u-shaped upper lip; drooling; and inability to make facial expressions, wrinkle the forehead, whistle, and/or difficulties with articulation of labial consonants.

In some cases of CFW, recruitment of other musculature can result in asymmetric synkinesis of facial and neck musculature. Most commonly, synkinesis affects the eye and facial muscles. During voluntary movement of the mouth, there may be involuntary eye closure, and during voluntary movement of the eye, there may be involuntary mouth or neck muscle movements [1].

In diagnosing CFW, it is important to establish that the facial weakness indeed was present at birth, as facial weakness may also be *acquired*, resulting from a different set of etiologies including infection (Bell’s palsy), neoplasia, or neurodegeneration.

## Differential diagnosis of congenital facial weakness

There is a broad differential for disorders with a prominent characteristic of CFW. This differential includes neurogenic, neuromuscular junction, myopathic, and other unknown or mixed causes (Fig. 1).

### Neurogenic causes of congenital facial weakness

There are a wide variety of neurogenic causes of congenital facial weakness including Athabasacan brain stem dysgenesis/Bosley-Salih-Alorainy syndrome**,** congenital fibrosis of the extraocular muscles (CFEOM) type 3A, CHARGE syndrome, hereditary congenital facial paresis, Moebius syndrome, and oculo-auriculo-vertebral spectrum (Fig. 1 and Table 1).

**Table 1:** Differential diagnosis for CFW disorders

Disorder Gene	Neurogenic CFW								
	Athabaskan brainstem dysgenesis syndrome/ Bosley-Salih-Alorainy syndrome	Congenital fibrosis of the extraocular muscles 3A with or without extraocular involvement^1^	CHARGE syndrome	Hereditary congenital facial paresis type 3	Moebius syndrome^2^	Oculo-auriculo-vertebral spectrum			
	*HOXA1*	*TUBB3*	*CHD7*	*HOXB1*	*PLXND1, REV3L*	*N/A*			
Mode of Inheritance	AR	AD	AD	AR	IC; AD (rare)	IC; AD (rare)			
Phenotype MIM #	#601536	#600638	#214800	#614744	%157900	%164210			
ORPHA #	69739/69737	45358	138	306530	570	141132			
Distinguishing Clinical Features Summary	Limited horizontal gaze and sensorineural hearing loss are the most common features; CFW in ~ 20% of cases. Other specific features include carotid artery anomalies, conotruncal heart defects, and central hypoventilation	CFEOM and CFW are clinical characteristics of TUBB3 disease caused by p.E410K, R262H, or D417H mutations. Additional features include developmental delay, progressive axonal sensorimotor polyneuropathy, Kallman syndrome, vocal cord paralysis, cyclic vomiting, and/or congenital joint contractures	Unilateral or bilateral CFW seen in ~ 40%. Common features include coloboma, microphthalmia, choanal atresia, cranial nerve dysfunction, ear anomalies (external ear abnormalities, ossicular malformations, Mondini defect of the cochlea, temporal bone abnormalities, and/or absent or hypoplastic semicircular canals)	Bilateral CFW (100%); Full ocular motility; Strabismus (42%); Sensorineural hearing loss (90%)	Congenital, nonprogressive facial weakness with limitation in abduction of one or both eyes. Associated features may include other cranial nerve involvement, strabismus, hearing loss, club foot, limb reduction defects, other limb anomalies, Poland anomaly, developmental delay, and autism. For most persons with Moebius syndrome, the etiology is unknown	CFP seen in ~ 10–45% of affected individuals. Characterized by facial asymmetry, preauricular or facial tags, ear malformations, microtia/anotia, and hearing loss			

Disorder gene	HPO Term	Neuromuscular CFW			Myopathic CFW					
		Congenital myasthenic syndrome 9 associated with acetylcholine receptor deficiency	Congenital myasthenic syndrome 10	Congenital myasthenic syndrome 11 associated with acetylcholine receptor deficiency	Carey-Fineman-Ziter syndrome	Central core disease/ Multiminicore disease		Centronuclear/myotubular myopathy		
		*MUSK*	*DOK7*	*RAPSN*	*MYMK*	*RYR1*	*SEPN1*	*BIN1*	*DNM2*	*MTM1*
Mode of Inheritance		AR	AR	AR	AR	AD, AR	AD, AR	AR	AD	XLR
Phenotype MIM #		#616325	#254300	#616326	#254940	#11700	#255310	#255200	#160150	#310400
ORPHA #		590	590	590	1358	597	2020	595	595	596
Distinguishing Clinical Features Summary		*MUSK* disease is characterized by early-onset muscle weakness with variable severity. Patients present with facial weakness, ptosis, ophthalmoplegia, episodic respiratory insufficiency and proximal muscle weakness	Clinical features of *DOK7* disease include limb-girdle pattern of weakness, waddling gait, ptosis, facial weakness, but rare is ophthalmoparesis	*RAPSN* disease may present in neonates. Some affected individuals have weakness confined to facial and masticatory muscles. Arthrogryposis multiplex congenita seems to be particularly common in infants with truncating *RAPSN* mutations	Clinical features include facial weakness, full ocular movements, upturned/broad nasal tip, micro/retrognathia, normal cognition, delayed motor milestones, and generalized muscle hypoplasia. Additional occasional signs and symptoms are congenital contractures, growth failure, feeding problems, ptosis, cleft palate, gastro/jejunostomy, thin tubular neck, pectoralis hypoplasia, hypoglossia, scoliosis, pulmonary hypertension, and/or cryptorchidism	Characterized by muscle weakness and hypotonia. Symptoms may range from mild to severe. In early-onset disease, newborns may have respiratory insufficiency, facial weakness, and poor suck		Pathogenic variants in *BIN1* have been described in a small group of individuals. Facial weakness may be present. The range of clinical severity is broad; presentation in infancy has been reported	Onset ranges from early (associated with severe disease) and late childhood or adulthood (associated with mild disease). Findings may include facial and extraocular muscle weakness, ptosis, and extremity weakness [Nance et al. 2012]. Of note, progressive weakness has been observed during the teen years and in adulthood in several individuals with a *DNM2* pathogenic variant	Severe disease manifests prenatally with polyhydramnios and decreased fetal movement; newborns have profound weakness, hypotonia, and respiratory failure that usually requires ventilatory support. Facial and extraocular muscles are often involved resulting in myopathic facies and ophthalmoparesis, respectively. Motor milestones are significantly delayed and most affected males fail to achieve independent ambulation

Disorder Gene	HPO Term	Myopathic CFW										
		Congenital myopathy- Zaharieva et al. [2016]^3^	Facioscapulohumeral muscular dystrophy		Myotonic dystrophy, type 1	Native American myopathy	Nemaline myopathy					*ZC4H2*-associated rare disorders
		*SCN4A*	*DUX4*	*SMCHD1*	*DMPK*	*STAC3*	*ACTA1*	*KLHL40*	*NEB*	*TPM2*	*TPM3*	*ZC4H2*
Mode of Inheritance		AR	AD	AD	AD	AR	AD, AR	AR	AR	AD	AD, AR	XLR
Phenotype MIM #		N/A	#158900	#158901	#160900	#255995	#161800	#615348	#256030	#609285	#609284	#314580
ORPHA #		N/A	269	269	273	168572	607	607	607	607	607	3454
Distinguishing Clinical Features Summary		Affected individuals from four families with compound heterozygous pathogenic variants in *SCN4A* were reported to have symptoms of a congenital myopathy, and all were noted to have mild to moderate facial weakness that was either present at birth (3 of 4 families) or developed during the first few days of life (1 family). Associated features included moderate to severe hypotonia, reduced muscle bulk, and neck, axial, and limb weakness	Characterized by progressive muscle weakness involving the face, scapular stabilizers, upper arm, lower leg, and hip girdle. Most individuals become symptomatic in their teens, but severe infantile onset with muscle weakness at birth is possible	This is a more rare type of FSHD, and is clinically indistinguishable from type 1 due to *DUX4* mutations	Congenital DM1 may include symptoms of CFW. Additional symptoms of affected neonates include hypotonia, positional malformations including club foot, and respiratory insufficiency	All reported patients had myopathic facies. Additional common features are congenital weakness, arthrogryposis, cleft palate, ptosis, short stature, kyphoscoliosis, club foot, and susceptibility to malignant hyperthermia provoked by anesthesia	The age of presentation of *ACTA1* NM ranges from severe congenital myopathy to later childhood onset. Facial weakness may be present	Findings of NM8 (*KLHL40*) include severe congenital lethal disease or fetal akinesia. Contractures, fractures, respiratory failure, and swallowing difficulties may be present at birth. CFW was identified in all 26 individuals studied by Ravenscroft et al. (2013)	The phenotype of NM2 (*NEB*) typically includes infants presenting in the first year of life with hypotonia, limb weakness, facial weakness, feeding difficulty, and respiratory weakness. The muscle disease is either static or slowly progressive such that most affected individuals survive to adulthood and live independently. Less common presentations are: death in utero due to fetal akinesia; severe hypotonia and weakness; facial weakness with a poor suck and swallow at birth; or predominantly distal weakness in older individuals	*TPM2* NM is generally associated with a typical congenital phenotype. The masticator (temporal) muscles and distal lower leg muscles are typically involved. CFW is typically present	*TPM3* NM may cause a severe congenital, intermediate congenital, or childhood onset phenotype. CFW is typically present	Fetal akinesia is generally noted prenatally. Clinical features include arthrogryposis, delayed motor development, facial and bulbar weakness, and skeletal abnormalities

Disorder Gene	HPO Term	Mixed/unknown CFW			
		Asymmetric crying facies^4^	*KAT6B* disorders	Marden-Walker syndrome^5^	Nablus mask-like facial syndrome
		22q11	*KAT6B*	*PIEZO2*	8q22.1
Mode of Inheritance		IC; AD	AD	IC; AD	AD
Phenotype MIM #		#125520	#603736	#248700	#608156
ORPHA #		1166	3047	2461	178303
Distinguishing Clinical Features Summary		Congenital hypoplasia of the depressor anguli oris muscle, congenital heart defects, microcephaly, intellectual impairment	Severe blepharophimosis, an immobile mask-like face, severe hypotonia and feeding problems, dislocated patellae, structural cardiac defects, pointed teeth, hypothyroidism, severe intellectual impairment	Mask-like face with blepharophimosis, micrognathia, cleft palate, low-set ears, kyphoscoliosis, joint contractures, Dandy-Walker malformation with hydrocephalus, vertebral abnormalities, intellectual impairment	Blepharophimosis, tight-appearing glistening facial skin, an abnormal hair pattern with an upswept frontal hairline, sparse arched eyebrows, flat and broad nose, long philtrum, distinctive ears, and a happy demeanor

^1^The name listed in OMIM is Congenital fibrosis of the extraocular muscles 3A with or without extraocular involvement; however, extraocular muscle involvement is required for the diagnosis

^2^Identification of potentially pathogenic variants in *PLXND1* or *REV3L* for Moebius syndrome is extremely rare

^3^Zaharieva et al. [2016], PMID=26700687

^4^Some cases of asymmetric crying facies are associated with 22q11 deletion

^5^Identification of potentially pathogenic variants in *PIEZO2* is extremely rare for Marden-Walker syndrome

Abbreviations: AD= autosomal dominant; AR= autosomal recessive; XLR= X-linked recessive; IC= isolated cases; N/A= not applicable; MIM= Online Mendelian Inheritance in Man; ORPHA= Orphanet; HPO= Human Phenotype Ontology

#### Athabascan brain stem dysgenesis syndrome (ABDS) (ORPHA:69739)/ Bosley-Salih-Alorainy syndrome (BSAS) (ORPHA:69737)

Athabascan brain stem dysgenesis syndrome (ABDS) and Bosley-Salih-Alorainy (BSAS) syndrome are allelic *HOX* gene disorders caused by recessive, pathogenic loss-of-function variants in the gene *HOXA1*. The *HOX* genes are homeodomain containing proteins that are critical for anterior–posterior differentiation in the developing embryo. In the developing vertebrate central nervous system, *HOX* genes function in hindbrain or rhombencephalon development. BSAS was first identified in Saudi Arabian and Turkish families [2, 3], while ABDS was first identified in Native American families [2, 4]. BSAS and ABDS are characterized by horizontal gaze palsy in which there is absent to markedly restricted ocular abduction and adduction. In some cases the horizontal gaze palsy is accompanied by retraction of the globe and narrowing of the palpebral fissure on attempted adduction, consistent with the diagnosis of Duane syndrome type 3. Most individuals with BSAS and ABDS also have bilateral sensorineural hearing loss caused by an absent cochlea and rudimentary inner-ear development and absent or hypoplastic carotid arteries with a corresponding absence of the carotid canal through which the artery normally passes. Approximately 20% of patients with BSAS or ABDS have congenital facial palsy [5]. Some affected individuals also have intellectual disability, autism spectrum disorder, moderate-to-severe central hypoventilation, swallowing difficulties, vocal cord paresis, conotruncal heart defects, macrocephaly, and malformations of inner ear bones and/or petrous bones. Individuals with simplex isolated Duane syndrome have not been found to harbor pathogenic variants in *HOXA1* [2].

#### Congenital fibrosis of the extraocular muscles 3A with or without extraocular involvement (CFEOM3A) (ORPHA:45358)

Congenital fibrosis of the extraocular muscles 3 (CFEOM3A) is a complex eye movement disorder with ptosis and restricted vertical and horizontal gaze with or without congenital facial palsy and non-ocular manifestations caused by a heterozygous, pathogenic missense variants in *TUBB3*, which encodes a beta-tubulin protein [6]. At least three specific *TUBB3* mutations are associated with CFEOM and CFP (c.1228G > A;p.Glu410Lys; c.785G > A;p.Arg262His; and c.1249G > C;p.Asp417His). Of these, the c.1228G > A;p.Glu410Lys variant is best defined, and the disorder caused by this specific mutation is known as the TUBB3 E410K syndrome. Features of the TUBB3 E410K syndrome (CFEOM3A, MIM #600638) include CFEOM, bilateral CFP, developmental delay, progressive sensorimotor polyneuropathy, Kallmann syndrome, stereotyped midface hypoplasia, and in some cases, vocal cord paralysis, tracheomalacia, and cyclic vomiting [7]. Patients with TUBB3 p.Arg262His and p.Asp417His mutations present with CFEOM, CFP, developmental delay, progressive sensorimotor polyneuropathy, and congenital joint contractures [6].

#### CHARGE syndrome (ORPHA:138)

CHARGE syndrome is a CFW syndrome due to heterozygous pathogenic variants in the gene *CHD7*, a chromatin helicase protein. CHARGE is a mnemonic for *c*oloboma, *h*eart defects, choanal *a*tresia, *r*etarded growth and development, *g*enital abnormalities, and *e*ar anomalies. 39% of *CHD7* mutation positive CHARGE syndrome patients were noted to have unilateral or bilateral CFW [8]. Other notable clinical features include additional cranial nerve dysfunction, impaired hearing, swallowing difficulties, hypogonadotropic hypogonadism, orofacial clefts, and/or tracheoesophageal fistula.

#### Hereditary Congenital Facial Paresis (HCFP) (ORPHA:306530)

Hereditary congenital facial paresis (HCFP) is a disorder with apparently isolated dysfunction of the facial nerve of germline genetic etiology. HCFP type 3 (HCFP3) results from recessive pathogenic loss-of-function variants in the *HOXB1* gene [9]. At present, 17 affected individuals from 6 separate families have been identified [9–13]. All 17 affected patients had bilateral CFW and were described as having masked-like facies. Eleven of 15 patients or 73% were noted to have sensorineural hearing loss (information unavailable for 2 patients). Seven of 17 or 41% of patients were noted to have esotropia. Patients had full ocular motility, and thus did not meet the minimum diagnostic criteria for Moebius syndrome (see below). Dysmorphic features included midface retrusion (16 of 17 patients or 94%), upturned nasal tip (13 of 17 patients or 76%), smooth philtrum (12 of 17 patients or 71%), and low-set ears (5 of 17 patients or 29%) [9–12]. Four affected members of one family harboring a homozygous *HOXB1* c.66C > G;p.Tyr22* mutation and 3 members of a family harboring a homozygous c.296_302del;p.Y99Wfs*20 mutation were noted to have external ear/auricular malformations; these external ear malformations have not been seen in the 4 other families who harbor different *HOXB1* mutations [9–11, 13].

Two additional genetic loci (HCFP1 (MIM %601471) and HCFP2 (MIM %604185) have been reported in large autosomal dominant pedigrees with incomplete penetrance [14–16]. Both loci are associated with unilateral or bilateral facial palsy; some individuals with HCFP2 also had hearing loss (a few cases associated with anomalous formation of the petrous portion of the temporal bone). Ocular movements are reported to be normal [14, 16].

#### Moebius syndrome (ORPHA:570)

Due to discrepancies and confusion in the literature, Moebius syndrome was defined at the Scientific Conference on Moebius Syndrome in 2007 as congenital, nonprogressive facial weakness with limited abduction of one or both eyes [17]. More recently, addition of the criterion of full ocular vertical motility to the minimum diagnostic criteria was suggested [18]. Given the diagnostic criteria, all persons with Moebius syndrome have CFW. The CFW may be unilateral or bilateral as well as partial or complete. Moebius subjects can present with additional clinical manifestations, which can include bilateral horizontal gaze palsy, other cranial nerve dysfunction, craniofacial and limb deformities (e.g. Poland syndrome and clubbed foot), mirror movements, sleep disorders, seizures, and neurocognitive and social impairments.

For the majority of cases of Moebius syndrome, the etiology is unknown. Both genetic and environmental etiologies have been proposed. Additionally, prenatal exposure to misoprostol and other agents has been known to be associated with a Moebius syndrome phenotype [19, 20]. De novo heterozygous missense *PLXND1* variants were reported in three individuals with Moebius syndrome and variable dysmorphic features (including microcephaly, epicanthal folds, flat nasal bridge, micrognathia, external ear defects, dental defects, clinodactyly, and low-set thumbs) [21]. However, one of these three individuals was noted to have a synonymous variant in *PLXND1* that is not predicted to alter splicing and, therefore, is presumed not to affect gene function. Additionally, three individuals were reported with de novo*,* heterozygous missense variants in *REV3L* (two with Moebius syndrome and one with isolated CFP without a defect in ocular motility), suggesting that HCFP and Moebius syndrome may be allelic disorders. Analysis of *Plxnd1* and *Rev3l* mutant mice revealed hypoplasia of the facial motor nucleus [21]. To date confirmation of additional cases of Moebius syndrome or CFW with mutations in these genes has not been reported.

#### Oculo-auriculo-vertebral spectrum (ORPHA:141132)

Oculo-auriculo-vertebral spectrum is a developmental disorder involving abnormal development of the first and second pharyngeal arches. Characteristic findings include facial asymmetry resulting from maxillary and/or mandibular hypoplasia; preauricular or facial tags; ear malformations such as microtia, anotia, or aural atresia; and hearing loss. The phenotype is heterogeneous and ranges from subtle asymmetries to frank, bilateral craniofacial involvement with involvement of other organ systems. Facial palsy (unilateral or bilateral involvement of either part or all branches of CN7) is observed in ~ 10–45% of affected individuals [22], and other cranial nerve abnormalities may also be seen. Other craniofacial malformations that may be present include cleft lip and/or palate. Non-craniofacial malformations involving skeletal, renal, and cardiac systems may be seen. The etiology in most cases is currently unknown.

### Neuromuscular junction causes of congenital facial weakness

Neuromuscular causes of congenital facial weakness include the congenital myasthenic syndromes, particularly types 9, 10, and 11 (Table 1).

#### Congenital myasthenic syndrome (CMS), types 9, 10, and 11 (ORPHA:590)

Congenital myasthenic syndromes are a group of disorders characterized by skeletal muscle weakness that worsens with exercise (myasthenia). The defect is due to recessive loss-of-function mutations in genes that encode proteins that function at the neuromuscular junction. While most congenital myasthenic syndromes may result in CFW, CFW is most typical in types 9, 10, and 11 [23–25]. Additional associated features may include ophthalmoplegia with congenital myasthenic syndrome, type 9 [26], respiratory muscle weakness with types 9, 10, and 11 [24, 27], and joint contractures and arthrogryposis, particularly with type 11. Treatment with cholinesterase inhibitors may be helpful for congenital myasthenic syndrome, type 9, treatment with cholinesterase inhibitors or amifampridine may be helpful for myasthenic syndrome, type 11 [27], and treatment with ephedrine may be helpful for congenital myasthenic syndrome type 10 [28].

### Myopathic causes of congenital facial weakness

CFW has been associated with both congenital myopathies and muscular dystrophies. The congenital myopathies with CFW include Carey-Fineman-Ziter syndrome, central core disease/multiminicore disease, centronuclear/myotubular myopathy, congenital myopathy reported by Zaharieva et al*.*, Native American myopathy, Nemaline myopathy, and *ZC4H2-*associated rare disorders. The muscular dystrophies include myotonic dystrophy, type 1 and infantile facioscapulohumeral muscular dystrophy (Table 1).

#### Carey-Fineman-Ziter syndrome (CFZS) (ORPHA:1358)

Carey-Fineman-Ziter syndrome, also known as congenital nonprogressive myopathy with Moebius sequence and Robin sequence, was first described by Carey et al*.* in 1982 in two siblings with Moebius sequence, Pierre Robin sequence, hypotonia, growth delay, and normal cognition. One sibling developed restrictive lung disease and died of pneumonia at age 37 years [29, 30]. CFZS results from recessive mutations in the *MYMK* gene [31]*. MYMK* encodes myomaker, a protein necessary for fusion of myoblasts to form multinucleated myofibers [31]. Compound heterozygous *MYMK* mutations were identified in the initial affected siblings reported by Carey et al*.*, as well as 6 additional affected individuals from 4 additional families. All 8 affected individuals had facial weakness, an upturned/broad nasal tip, micro/retrognathia, delayed motor milestones, generalized muscle hypoplasia, and normal cognition. Some also had downslanting palpebral fissures, epicanthal folds, flat nasal root, and mandibular hypoplasia. None had an abducens nerve palsy, a required feature for the diagnosis of Moebius syndrome [31].

#### Central core disease (ORPHA:597)/multiminicore disease (ORPHA:2020)

Central core disease and multiminicore disease may be caused by pathogenic variants in *RYR1* or *SEPN1*. Central core disease is named due to the presence of disorganized areas of ‘central cores’ seen on muscle biopsy, while multiminicore is named due to disorganized ‘minicores’.

Mutations in *RYR1* may cause autosomal recessive or autosomal dominant central core disease, autosomal recessive minicore myopathy with external ophthalmoplegia, and autosomal recessive or autosomal dominant neuromuscular disease, congenital, with uniform type 1 fiber. *RYR1* encodes the skeletal muscle ryanodine receptor, which is a ligand-gated calcium channel important in calcium signaling in the sarcoplasmic reticulum. For autosomal dominant central core disease, onset is typically at birth or early childhood with features of facial weakness and nonprogressive limb weakness, or in early childhood with nonprogressive limb weakness and hypotonia. Affected patients generally survive to adulthood. Multiminicore myopathy due to recessive mutations in *RYR1* presents generally in infancy with external ophthalmoplegia, limb weakness, and wasting of hip girdle muscles similar to central core disease [32]. Additional features include bulbar, facial, or respiratory weakness, and joint abnormalities including hyperlaxity, contractures, and arthrogryposis. Rare cases of autosomal recessive inheritance, with homozygous or compound heterozygous *RYR1* mutations have been described presenting with severe congenital ophthalmoplegia and facial weakness in the setting of only mild skeletal myopathy. These patients were susceptible to malignant hyperthermia [33].

Mutations in the selenoprotein N gene (*SEPN1)* have been identified in individuals with multiminicore disease, muscular dystrophy with rigid spine, and congenital myopathy with fiber-type disproportion. Homozygous or compound heterozygous mutations in *SEPN1* cause autosomal recessive multiminicore disease. Multiminicore disease is broadly classified into four groups: classic form (75% of individuals); moderate form with hand involvement (< 10%); antenatal form with arthrogryposis multiplex congenita (< 10%); and ophthalmoplegic form (< 10%) [34]. Onset of the classic form is usually at birth or early in childhood with hypotonia, delayed motor milestones, scoliosis, and significant respiratory involvement. Most patients with multiminicore disease and *SEPN1* mutations exhibit a variable degree of facial weakness [34, 35].

#### Centronuclear (ORPHA:595, 596)/ myotubular myopathy

Centronuclear/myotubular myopathy may be caused by homozygous or compound heterozygous pathogenic variants in the gene *BIN1*, heterozygous pathogenic variants in the gene *DNM2,* or hemizygous pathogenic variants in *MTM1*. Muscle biopsy in these conditions reveals small, rounded myofibers with increased percentages of centrally located nuclei. Pathogenic variants in *BIN1* have been described in a small group of individuals. *BIN1* encodes amphiphysin-2, which binds to DNM2 during clathrin-dependent endocytosis [36]. The range of clinical severity is broad, and CFW has been reported.

*DNM2* encodes dynamin-2-protein, a large GTPase protein involved in clathrin-dependent and clathrin-independent endocytosis and intracellular membrane trafficking. For *DNM2* centronuclear/myotubular myopathy, the range of symptoms in affected individuals is broad, and severe disease may present with CFW. Findings may include facial and extraocular muscle weakness, ptosis, and extremity weakness [36].

*MTM1* encodes myotubularin, a tyrosine phosphatase protein required for muscle cell differentiation. Myopathy due to *MTM1* mutations is also known as X-linked centronuclear myopathy (CNMX). In CNMX, muscle weakness ranges from mild to severe (classic disease). The muscle disease of CNMX is not obviously progressive. CFW may be present in severe/classic disease. Female carriers of CNMX are generally asymptomatic.

#### Congenital myopathy- Zaharieva et al. [2016]

Recessive loss of function homozygous or compound heterozygous variants in *SCN4A*, which encodes sodium voltage-gated channel, alpha subunit 4, have been reported to cause a spectrum of disease ranging from severe fetal hypokinesia to a ‘classical’ congenital myopathy. Affected individuals from four families with compound heterozygous pathogenic variants in *SCN4A* were reported to have symptoms of a congenital myopathy, and all were noted to have mild to moderate facial weakness that was either present at birth (3 of 4 families with CFW) or developed during the first few days of life (1 family). Associated features included moderate to severe hypotonia, reduced muscle bulk, and neck, axial, and limb weakness [37].

#### Facioscapulohumeral muscular dystrophy (FSHD) (ORPHA:269)

Facioscapulohumeral muscular dystrophy (FSHD) type 1 is an autosomal dominant condition caused by mutations in *DUX4* [38]. FSHD typically presents before age 20 years with weakness of the facial muscles and the stabilizers of the scapula or the dorsiflexors of the foot. There is extreme clinical variability. In some cases, CFW may be present. In FSHD, the muscle weakness is slowly progressive and approximately 20% of affected individuals eventually require a wheelchair. Life expectancy is not shortened. The incidence is approximately 4 individuals affected per 100,000 people [38].

Although some controversy remains, FSHD is likely caused by inappropriate expression of the double homeobox-containing gene *DUX4* in muscle cells. *DUX4* lies in the macrosatellite repeat D4Z4 on chromosome 4q35, which has a length between 11 and 100 repeat units on normal alleles. Approximately 95% of individuals with FSHD have a D4Z4 allele of between one and ten repeat units. The shortening of the D4Z4 allele causes chromatin relaxation at the D4Z4 locus and *DUX4* promoter and thereby derepression of *DUX4*. This common form of FSHD is designated facioscapulohumeral muscular dystrophy 1 (FSHD1). Molecular genetic testing measures the length of the D4Z4 allele [38].

#### Myotonic dystrophy, type 1 (DM1) (ORPHA:273)

Myotonic dystrophy type 1 (DM1) is a multisystem disorder that affects skeletal and smooth muscle as well as the ocular, cardiovascular, endocrine, and central nervous systems. The clinical findings, which span a continuum from mild to severe, have been categorized into three overlapping phenotypes: mild, classic, and congenital. Congenital DM1 is characterized by hypotonia and severe generalized weakness at birth, including CFW. Affected infants often have an inverted V-shaped upper lip, which is characteristic of significant facial diplegia. Before birth polyhydramnios and reduced fetal movement may be noted. Positional malformations such as club foot and respiratory insufficiency leading to early death may also occur. Intellectual disability is common.

DM1 is caused by expansion of a CTG trinucleotide repeat in the non-coding region of *DMPK*. A normal allele has 5–34 CTG repeats, a premutation allele has 35–49 CTG repeats, and a full penetrance allele has ≥ 50 CTG repeats. Congenital DM1 is associated with a repeat size of > 1000 CTG repeats. Generally, CTG repeat expansion causing congenital DM1 occurs when the mother is transmitting the abnormal allele [39].

#### Native American myopathy (NAM) (ORPHA:168572)

Native American myopathy is an autosomal recessive condition originally identified in the Lumbee Native American population of North Carolina, which presents with features of congenital onset of muscle weakness, susceptibility to malignant hyperthermia, multiple joint contractures, ptosis, and dysmorphic features. All reported patients had myopathic facies and hypotonia and generalized weakness present from birth. Muscle biopsies of affected individuals reveal a non-specific myopathic pattern. Affected Native American individuals with Native American myopathy were identified to have a homozygous *STAC3* pathogenic variant, c.1046G > C;p.Trp284Ser [40].

Subsequently Native American myopathy also has been identified in individuals of non-Lumbee Native American descent and additional pathogenic variants other than the p.Trp284Ser variant have now been identified [41–43]. Native American myopathy has overlapping features with Moebius and Carey-Fineman-Ziter syndromes [42].

#### Nemaline myopathy (ORPHA:607)

Nemaline myopathy (NM) is named for the finding on muscle biopsy of abnormal rod-like structures within muscle cells termed nemaline bodies [44]. It can be caused by pathogenic variants in multiple genes, including *ACTA1*, *KLHL40*, *NEB*, *TPM2*, and *TPM3*.

*ACTA1* encodes the skeletal muscle protein alpha-actin, which forms the core of the thin filament of the sarcomere that functions in muscle contraction. Biallelic pathogenic variants in *ACTA1* cause autosomal recessive NM; heterozygous pathogenic variants cause autosomal dominant NM3. *ACTA1* disease causes 20–25% of all nemaline myopathy, but 50% of severe nemaline myopathy. The age of presentation ranges from severe congenital myopathy to later childhood onset. Facial weakness may be present.

*KLHL40*, or kelch-like family member 40, is a protein that is necessary for sarcomere structure and contractility [45]. Biallelic pathogenic variants in *KLHL40* cause autosomal recessive nemaline myopathy 8. Findings are severe congenital lethal disease or fetal akinesia. In addition to severe fetal akinesia or hypokinesia, contractures, fractures, respiratory failure, and swallowing difficulties at birth, facial weakness was present at birth in all 26 individuals studied by Ravenscroft et al. [46].

*NEB* encodes nebulin, a cytoplasmic matrix protein in skeletal muscle. Biallelic pathogenic variants in *NEB* cause autosomal recessive, nemaline myopathy 2, which accounts for about 50% of cases of nemaline myopathy. The phenotype of the typical congenital form includes presentation within the first year of life with hypotonia, limb weakness, facial weakness, feeding difficulty, and respiratory weakness. The muscle disease is either static or slowly progressive such that most affected individuals survive to adulthood and live independently. Less common presentations are: death in utero due to fetal akinesia; severe hypotonia and weakness; CFW with a poor suck and swallow at birth; or predominantly distal weakness in older individuals [44].

*TPM2* encodes beta-tropomyosin, a component of the thin filament of the sarcomere, and is primarily expressed in slow, type 1 skeletal muscle fibers. Heterozygous pathogenic variants in *TPM2* cause autosomal dominant nemaline myopathy 4 which accounts for < 1% of cases of nemaline myopathy. *TPM2* nemaline myopathy is generally causative of a typical congenital phenotype and is commonly associated with involvement of the masticator muscles and distal lower leg muscles. Affected patients typically have a myopathic face with facial diplegia.

*TPM3* encodes alpha-tropomyosin, another thin filament component of the sarcomere. Biallelic pathogenic variants and heterozygous pathogenic variants in *TPM3* cause autosomal recessive or autosomal dominant nemaline myopathy, 1, respectively which accounts for ~ 2–3% of cases of nemaline myopathy. *TPM3* disease phenotypic range includes severe congenital (autosomal recessive; ~ 16% of cases), intermediate congenital, or childhood onset (autosomal dominant) types. Findings include weakness that is predominantly proximal and generalized with or without facial weakness; hypotonia and depressed deep tendon reflexes, with preserved sensation and normal cognition; and feeding difficulties related to facial and bulbar weakness, respiratory difficulties, recurrent infections or a weak cough related to restrictive lung disease from weakness of the respiratory muscles [44].

#### ZC4H2-associated rare disorders (ZARD) (ORPHA:3454)

*ZC4H2*-associated rare disorders includes a spectrum of disease with arthrogryposis multiplex and peripheral and central nervous system involvement [47]. This spectrum includes Wieacker-Wolff syndrome (WRWF), an X-linked recessive condition caused by pathogenic variants in *ZC4H2*. *ZC4H2* encodes a zinc-finger protein, expressed in a variety of tissues. Boys with WRWF generally had decreased fetal movements in utero and are born with severe contractures. Additional clinical findings include delayed motor development, CFW, bulbar weakness, characteristic dysmorphic facial features (carp-like mouth, narrow palate, micrognathia, long philtrum, upturned nares, and short neck), skeletal abnormalities such as hip dislocation, scoliosis, pes equinovarus, intellectual disability, and Duane syndrome. Carrier females may exhibit mild features of the disorder due to skewed X-chromosome inactivation [48].

### Other, Including Unknown or Mixed Causes of Congenital Facial Weakness

There are other causes of CFW that are of other unknown or mixed etiology. These types of CFW include asymmetric crying facies, *KAT6B* disorders, Marden-Walker syndrome, and Nablus mask-like facial syndrome.

#### Asymmetric crying facies (ACF) (ORPHA:1166)

Asymmetric crying facies (ACF) describes newborns with unilateral facial weakness noticed when the infant cries. Primary (myopathic) or secondary (neurogenic) agenesis or hypoplasia of the depressor anguli oris muscle may cause developmental forms of asymmetric crying facies, which is also known as congenital unilateral lower lip palsy (CULLP). ACF may be associated with heart defects and/or deletion 22q11 [49].

#### KAT6B disorders (ORPHA:3047)

*KAT6B* disorders include Say-Barber-Biesecker-Young-Simpson syndrome (SBBYSS), characterized by distinctive facial features including severe blepharophimosis, an immobile, mask-like face, a bulbous nasal tip, and a small mouth with a thin upper lip; neurologic findings including severe hypotonia and feeding problems in infancy, severe intellectual disability, delayed motor milestones, and significantly impaired speech; and skeletal findings including joint laxity, abnormally long thumbs and great toes, and dislocated or hypoplastic patellae. The mask-like facial appearance is due to bilateral CFW. Other common findings are congenital heart defects (~ 50%), dental anomalies (small and pointed teeth), and abnormalities of thyroid structure or function. To date, most individuals with a *KAT6B*-related disorder have had a de novo dominant pathogenic variant in the *KAT6B* gene, which encodes a histone acetyltransferase [50].

#### Marden-Walker syndrome (MWKS) (ORPHA:2461)

Marden-Walker syndrome **(**MWKS) is characterized by “immobile facies,” blepharophimosis, joint contractures including camptodactyly, scoliosis, cleft palate, diminished muscular bulk, developmental delay, and hindbrain malformations, most commonly Dandy-Walker malformation. For the majority of cases, the etiology is unknown. One patient with MWKS has been identified with a de novo missense mutation (p.R2686C) in *PIEZO2.* Mutations in *PIEZO2* were also found in the phenotypically overlapping syndromes, Gordon syndrome or distal arthrogryposis, type 3 (DA3) and distal arthrogryposis, type 5 (DA5) [51].

#### Nablus mask-like facial syndrome (ORPHA:178303)

Nablus mask-like facial syndrome is a contiguous gene deletion syndrome at chromosome 8q22.1 characterized by blepharophimosis, tight-appearing glistening facial skin, an abnormal hair pattern with an upswept frontal hairline, sparse arched eyebrows, flat and broad nose, long philtrum, distinctive ears, and a happy demeanor [52–54].

### Evaluation strategy for consultants

Establishing a specific cause of congenital facial weakness enables accurate discussions of treatment, management, and prognosis and allows for precise genetic counseling. Consultations generally begin with a full medical history (including family history) and physical examination. Family history assessment includes an extended pedigree with attention to relatives with related craniofacial findings and documentation of relevant findings through direct examination or review of medical records, including results of molecular genetic testing. Depending on the diagnosis, genetic testing may be indicated (Table 2).

**Table 2:** Evaluation and examination recommended for patients with congenital facial weakness

**For all patients with CFW:**		
Dysmorphology exam by geneticist	Assess for dysmorphic features Obtain family history Assess for intrauterine exposures	If facial asymmetry/hemifacial microsomia ± microtia present, consider a diagnosis of oculo-auriculo-vertebral spectrum disorder and order renal sonogram and cervical x-ray If characteristic CHARGE ear present, consider a diagnosis of CHARGE syndrome If ear anomalies are present, consider HCFP3 If Pierre-Robin sequence, cleft palate, scoliosis, and/or contractures are present, consider Carey-Fineman-Ziter or fetal akinesia syndromes If personal or family history of malignant hyperthermia, consider Native American myopathy or *RYR1* disease If a specific diagnosis is suspected that is caused by a single gene or few genes, especially in the case of positive family history, order sequencing with deletion/duplication analysis for the suspected genes or D4Z4 allele contraction testing in the case of facioscapulohumeral muscular dystrophy When a specific diagnosis caused by a single gene is not suspected, chromosome analysis and chromosomal microarray may be offered. Consider whole exome sequencing
Neurology exam	Full cranial nerve examination including testing for anosmia and hearing Assess for presence of peripheral neuropathy and mirror movements Assess for muscle weakness and signs of myopathy or myotonia	Brainstem/cranial nerve/orbital MRI if considering diagnosis of Moebius syndrome or HCFP to assess for absence or hypoplasia of CN7 and other cranial nerves (1, 3, 6, 8–11). MRI may reveal abnormalities of the corpus callosum, pituitary gland, olfactory sulci and olfactory bulbs in individuals with TUBB3 E410K EMG, nerve conduction studies to narrow differential Consider EEG to rule out seizures Consider muscle biopsy if limb muscles are abnormal Consider sleep studies depending on history If weakness of shoulder girdle, consider diagnosis of facioscapulohumeral muscular dystrophy Isolated congenital lower lip palsy is suggestive of asymmetric crying facies Peripheral neuropathy may be seen with CFEOM3
Ophthalmology exam	Assess extraocular movements and for presence of ptosis Assess for aberrant movements such as globe retraction found in Duane syndrome (associated with ABDS/BSAS)	If abduction deficit or bilateral horizontal gaze palsy with full vertical motility, consider diagnosis of Moebius syndrome or ABDS/BSAS Ophthalmoplegia and ptosis may be seen with CFEOM3, CMS9, and multiminicore myopathy
**For some patients with CFW:**		
ENT referral	Assess for possible hearing loss and temporal bone/ear anomalies	CT to assess for temporal bone abnormalities and/or hypoplastic semicircular canals in the case of CHARGE syndrome and ABDS/BSAS
Cardiology referral	Assess for congenital heart defect (associated with BSAS/ABDS, oculo-auriculo-vertebral spectrum disorder, and Moebius syndrome)	Echocardiogram
Endocrinology referral	Assess for hypogonadotropic hypogonadism/ Kallmann syndrome (associated with CFEOM3 and CHARGE)	Hormone testing Smell testing
Developmental pediatrics referral	Assess for developmental delays	PT, OT, and ST as indicated
Orthopedics/Physical Medicine and Rehabilitation referral	Assess for scoliosis, limb length discrepancy, and need for orthotics	X-rays
Craniofacial Team/Dental referral	Assess for orofacial clefting, Pierre Robin sequence, palatal and tongue defects, dental anomalies	Craniofacial exam by a craniofacial surgeon Dental exam

The medical history and physical examination are directed at identifying features associated with specific causes of congenital facial weakness (Table 1, Additional file 1). The consultant should obtain detailed information regarding the onset and severity of the CFW and the presence of additional symptoms. Past medical history, including surgical history, should be obtained with special attention to any incidence of malignant hyperthermia. A genetics evaluation should include dysmorphology assessment that may aid in narrowing the diagnosis. A full cranial nerve examination should be completed as well as an examination for signs and symptoms of primary muscle disease (preferably by a neurologist or neuromuscular specialist). Ocular motility should be assessed (preferably by an ophthalmologist or orthoptist). Patients should receive audiology evaluation as well as a smell test to assess for anosmia. Endocrine evaluation should be performed if there are signs of delayed puberty, growth failure, or thyroid abnormalities. A developmental assessment should be performed if developmental delays are suspected, and feeding or speech therapy and other supportive services may be offered to help with physical and social impairments and improve the quality of life of affected individuals. Additional work-up may include assessment for congenital heart defect, skeletal defects, dental or craniofacial anomalies, or other system involvement, guided by associated findings.

CFP with or without strabismus, but without a limitation in horizontal or vertical ocular movement and without other major systemic involvement is suggestive of HCFP and includes the *HOXB1* syndrome as well as genetically undefined forms of autosomal dominant CFP (HCFP1, HCFP2). Impairment in ocular abduction, with full ocular vertical mobility, in association with CFW suggests a diagnosis of Moebius syndrome [18] or *HOXA1* syndromes. CFP with both horizontal and vertical gaze limitations and ptosis (i.e. CFEOM) may be suggestive of *TUBB3* syndromes and should prompt further evaluations for associated peripheral axonal polyneuropathy, endocrine work-up for Kallmann syndrome, and neurocognitive testing. CFP in combination with a coloboma, deafness, and/or a distinctive ear appearance is suggestive of CHARGE syndrome. CFW in association with facial asymmetry, microtia, preauricular tags or pits, micrognathia, aural atresia, or conductive hearing loss is suggestive of oculo-auriculo-vertebral spectrum disorder. Isolated congenital lower lip palsy is consistent with a diagnosis of asymmetric crying facies. If history of malignant hyperthermia is present, a diagnosis of Native American Myopathy or other congenital myopathies like *RYR1* should be considered.

MRI of cranial nerves may reveal absence or hypoplasia of the facial nerve. Axial and sagittal oblique T2-SPACE MRI series is recommended for evaluation of the facial nerve. MRI of facial muscles may reveal absence or hypoplasia. Muscle ultrasound and MRI may help differentiate amongst the congenital myopathies based on the distribution of affected muscle groups. Additionally, electromyogram may aid in the diagnosis of myasthenia or a congenital myopathy as well as help identify an appropriate site for muscle biopsy. Nerve conduction studies may also be used to help narrow the differential. Creatine kinase levels may be elevated in cases of myopathy.

### Genetic testing

Molecular genetic testing approaches may include a combination of gene-targeted testing (multi-gene panel or single-gene testing) and genomic testing (comprehensive genomic sequencing or chromosomal microarray analysis [CMA]). Gene-targeted testing requires the clinician to narrow in on a particular diagnosis and hypothesize which gene(s) are likely involved. Chromosomal microarray (CMA) may be considered in patients with distinguishing, multiple phenotypic features suggestive of a contiguous gene deletion syndrome. Whole exome sequencing (WES) and whole-genome sequencing (WGS) may be considered if the phenotype alone is insufficient to support gene-targeted testing. In some instances where the genetic etiology for a particular diagnosis is currently unknown, genetic testing may not be indicated at this time.

### Conclusions

The differential diagnosis for congenital facial weakness conditions is quite broad and extensive. The clinician must be knowledgeable regarding the many neurogenic, neuromuscular junction, and myopathic causes of congenital facial weakness as well as other unknown or mixed causes to make an accurate diagnosis. Medical history, physical examination, and additional testing as indicated are necessary to narrow in on the correct diagnosis. Depending on the cause of the congenital facial weakness, confirmatory genetic testing may be available.

## Supplementary Information

**Additional file 1**. Clinical Features of CFW Disorders.

## Data Availability

Not applicable.

## Abbreviations

ABDS: Athabascan brain stem dysgenesis
ACF: Asymmetric crying facies
BSAS: Bosley-Salih-Alorainy syndrome
CFEOM: Congenital fibrosis of the extraocular muscles
CFP: Congenital facial paralysis
CFW: Congenital facial weakness
CFZS: Carey-Fineman-Ziter syndrome
CMS: Congenital myasthenic syndrome
CN7: Cranial nerve 7
DM1: Myotonic dystrophy, type 1
FSHD: Facioscapulohumeral muscular dystrophy
HCFP: Hereditary congenital facial paresis
MWKS: Marden-Walker syndrome
NAM: Native American myopathy
SBBYSS: Say-Barber-Biesecker-Young-Simpson syndrome
CNMX: X-linked centronuclear myopathy
ZARD: *ZC4H2*-Associated rare disorders
